# Regional and temporal variations in coding of hospital diagnoses referring to upper gastrointestinal and oesophageal bleeding in Germany

**DOI:** 10.1186/1472-6963-11-193

**Published:** 2011-08-17

**Authors:** Ingo Langner, Rafael Mikolajczyk, Edeltraut Garbe

**Affiliations:** 1 Bremen Institute for Prevention Research and Social Medicine, Bremen University, Achterstraße 30, D-28359 Bremen, Germany

## Abstract

**Background:**

Health insurance claims data are increasingly used for health services research in Germany. Hospital diagnoses in these data are coded according to the International Classification of Diseases, German modification (ICD-10-GM). Due to the historical division into West and East Germany, different coding practices might persist in both former parts. Additionally, the introduction of Diagnosis Related Groups (DRGs) in Germany in 2003/2004 might have changed the coding. The aim of this study was to investigate regional and temporal variations in coding of hospitalisation diagnoses in Germany.

**Methods:**

We analysed hospitalisation diagnoses for oesophageal bleeding (OB) and upper gastrointestinal bleeding (UGIB) from the official German Hospital Statistics provided by the Federal Statistical Office. Bleeding diagnoses were classified as "specific" (origin of bleeding provided) or "unspecific" (origin of bleeding not provided) coding. We studied regional (former East versus West Germany) differences in incidence of hospitalisations with specific or unspecific coding for OB and UGIB and temporal variations between 2000 and 2005. For each year, incidence ratios of hospitalisations for former East versus West Germany were estimated with log-linear regression models adjusting for age, gender and population density.

**Results:**

Significant differences in specific and unspecific coding between East and West Germany and over time were found for both, OB and UGIB hospitalisation diagnoses, respectively. For example in 2002, incidence ratios of hospitalisations for East versus West Germany were 1.24 (95% CI 1.16-1.32) for specific and 0.67 (95% CI 0.60-0.74) for unspecific OB diagnoses and 1.43 (95% CI 1.36-1.51) for specific and 0.83 (95% CI 0.80-0.87) for unspecific UGIB. Regional differences nearly disappeared and time trends were less marked when using combined specific and unspecific diagnoses of OB or UGIB, respectively.

**Conclusions:**

During the study period, there were substantial regional and temporal variations in the coding of OB and UGIB diagnoses in hospitalised patients. Possible explanations for the observed regional variations are different coding preferences, further influenced by changes in coding and reimbursement rules. Analysing groups of diagnoses including specific and unspecific codes reduces the influence of varying coding practices.

## Background

Health insurance claims data are increasingly used for health services research in Germany [1-8]. Such studies frequently focus on the main hospital discharge diagnosis as the relevant outcome, and their validity depends fundamentally on the quality of coding of those diagnoses. Studies assessing the internal and external validity of hospital diagnoses recorded in German claims data are rare [9].

Hospital discharge diagnoses in Germany are coded according to the International Classification of Diseases 10^th ^Revision, German modification (ICD-10 GM) [10].

In the context of a project investigating the risk of bleeding in patients treated with anticoagulants [11,12], we assessed the coding of hospitalisation diagnoses for serious bleeding events of the upper gastrointestinal tract by analysing the regional and time variation of their incidence in Germany. For upper gastrointestinal bleeding (UGIB) and oesophageal bleeding (OB), ICD-10 GM allows the coding of unspecific clinical conditions (e.g. haematemesis) and the more specific coding of the underlying disease (e.g. oesophageal variceal bleeding) (Table 1). Detailed mandatory coding rules for hospital diagnoses have been introduced by the Institute for the Hospital Remuneration System (INEK) in Germany since 2002 [13]. According to these rules, patients admitted for UGIB in whom ulcers, erosions or oesophageal varices are detected during endoscopy receive as diagnosis the specific lesion including bleeding, even if no bleeding was observed during the endoscopic investigation or during the hospital stay. In this case, it is assumed that the bleeding event before hospitalisation can be ascribed to the detected lesion. Only if the cause of bleeding cannot be identified, an unspecific code referring to bleeding as a clinical condition should be used. Despite these rules, regional or temporal variations in coding might still exist. The historical separation of East and West Germany may have influenced coding practices. Further, the introduction of Diagnosis Related Group (DRG) based payment for hospital services in Germany may have affected the coding due to changed reimbursement rules. The DRG system was initiated in Germany on a voluntary basis in 2003 and became compulsory from 2004 onwards. Several changes of the DRG system occurred between 2004 and 2005 [14,15]. In the 2004 DRG system, the additional coding of a procedure such as a gastroscopy in a patient with OB or UGIB could have led to a grouping into a DRG with a lower reimbursement ("reduced reimbursement while providing additional service", [15]). This was changed in the 2005 DRG system and such change could have had an impact on coding practices [16].

**Table 1:** ICD-10-GM Codes for Oesophageal and Upper Gastrointestinal Bleeding Categorised by Diagnosis Subgroup

Diagnosis group	Diagnosis subgroup regarding type of coding	Codes	Description of coded diseases
Oesophageal	unspecific	K92.0	Haematemesis
	
bleeding	specific	I85.0	Oesophageal varices with bleeding
		K22.6	Mallory-Weiss Syndrome,
		K22.8	Other specified diseases of oesophagus

Upper	unspecific	K92.2	Gastrointestinal haemorrhage, unspecified
	
gastrointestinal bleeding	Specific	K25.0	Gastric ulcer, acute with haemorrhage
		K25.2	Gastric ulcer, acute with both haemorrhage and perforation
		K25.4	Gastric ulcer, chronic or unspecified with haemorrhage
		K25.6	Gastric ulcer, chronic or unspecified with both haemorrhage and perforation
		K26.0	Duodenal ulcer, acute with haemorrhage
		K26.2	Duodenal ulcer, acute with both haemorrhage and perforation
		K26.4	Duodenal ulcer, chronic or unspecified with haemorrhage
		K26.6	Duodenal ulcer, chronic or unspecified with both haemorrhage and perforation
		K27.0	Peptic ulcer, site unspecified, acute with haemorrhage
		K27.2	Peptic ulcer, site unspecified, acute with both haemorrhage and perforation
		K27.4	Peptic ulcer, site unspecified, chronic or unspecified with haemorrhage
		K27.6	Peptic ulcer, site unspecified, chronic or unspecified with both haemorrhage and perforation
		K28.0	Gastrojejunal ulcer, acute with haemorrhage
		K28.2	Gastrojejunal ulcer, acute with both haemorrhage and perforation
		K28.4	Gastrojejunal ulcer, chronic or unspecified with haemorrhage
		K28.6	Gastrojejunal ulcer, chronic or unspecified with both haemorrhage and perforation
		K29.0	Acute haemorrhagic gastritis

In this study, we investigated regional and temporal variations in coding practices for OB and UGIB using the official German Hospital Statistics (GHS) database.

## Methods

### Data Source and Variables

We analysed data from the official GHS database for the years 2000-2005 as provided by the Research Data Centres of the Federal Statistical Office. The GHS database is a register of all in-patient treatments in German hospitals and includes information on age, sex, place of residence (on district level, in total 469 different districts), date of hospital admission and discharge, and the main discharge diagnosis for each hospitalisation event. According to the German coding system, the main discharge diagnosis is defined as the diagnosis which after considering all clinical findings was determined as the main cause of the hospitalisation [13]. These diagnoses are coded according to the German modification of ICD-10, which is updated on an annual basis [10]. Only hospitalisation events where the patient's place of residence was in Germany were included in our analyses. Use of this data is regulated by German law. It requires a contract with the data holder agency (Federal Statistical Office) which is responsible for the compliance with data protection regulations. Access to the data is permitted only by means of teleprocessing. We prepared and transmitted a statistical analysis program written in SAS (SAS 8.02 software: SAS Institute, Cary, NC). The SAS-program was executed in the research data centre of the Federal Statistical Office and the results were sent back to our institute. We investigated two groups of diagnoses: oesophageal bleeding (OB) and upper gastrointestinal bleeding (UGIB). The respective ICD-10-GM codes are shown in Table 1. These codes were valid throughout the study period from 2000 to 2005. Coding rules regarding specific and unspecific bleeding were introduced in 2002 and remained unchanged during the study period [13]. For the analysis of regional differences between the former East and West Germany, we used patients' place of residence (assuming that most of the admissions will be in the region of residence). Administrative districts of the federal states Brandenburg, Mecklenburg-Western Pomerania, Saxony, Saxony-Anhalt, and Thuringia were classified as East and of Schleswig-Holstein, Hamburg, Lower Saxony, Bremen, North-Rhine-Westphalia, Hesse, Rhineland-Palatinate, Baden-Wuerttemberg, Bavaria, and Saarland as West. Berlin was not included as it can not be unequivocally assigned to either former East or West Germany.

In addition to the GHS database, we obtained information from the German Population Statistics and German Area Statistics provided by the German Federal Statistical Office and calculated population density (PD) per district as population number per square kilometre. The continuous measure of PD was categorized into tertiles (low PD = less then 138; intermediate PD = 138 to less then 373; high PD = 373 and more inhabitants per km^2^) and was used as a proxy for urbanity. We speculated that unspecific coding can be more common in hospitals in more rural areas and thus adjusting for population density would control this bias.

### Statistical Analysis

Incidence rates of hospitalisations for OB and UGIB were calculated as the number of hospitalisations with the corresponding codes in the GHS database per 100,000 persons in the population per sex and year. 95% confidence intervals were obtained from the Poisson distribution. Furthermore, we assessed the proportion of unspecific diagnoses among all diagnoses for OB or UGIB, respectively. Corresponding 95% confidence intervals (95% CI) were calculated following the method recommended by Newcombe & Altman [17]. East-West differences in specific, unspecific and overall hospitalisation diagnoses were estimated as incidence ratios, using log-linear regression models adjusting for sex, age (in 5-year age groups) and population density (in three categories) at the cases' residence. Initially, Poisson regression models were used, but since there was an indication of overdispersion, we applied a negative binomial distribution [18,19] for the response. All regression analyses were stratified by year. Non-overlapping 95% confidence intervals were interpreted as a significant difference. All analyses were done using SAS version 8.02 (SAS Institute, Cary, NC).

## Results

**Figure 1:**
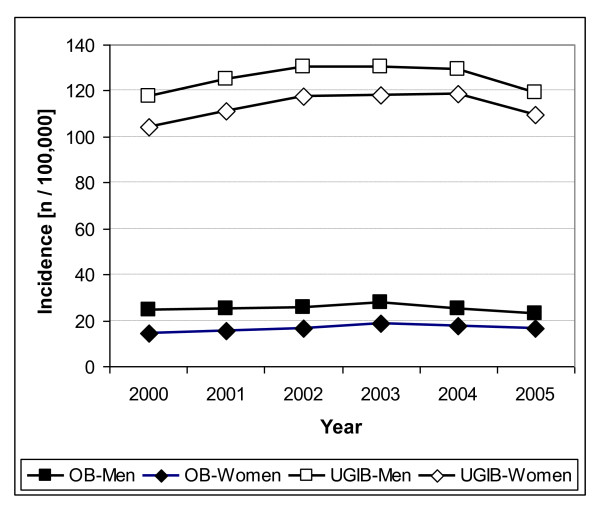
**Incidence of Gastrointestinal Bleeding Diagnoses by Sex and Year**. Annual sex-specific incidence of hospitalisations due to upper gastrointestinal bleeding (UGIB) or oesophageal bleeding (OB) per 100,000 population.

**Figure 2:**
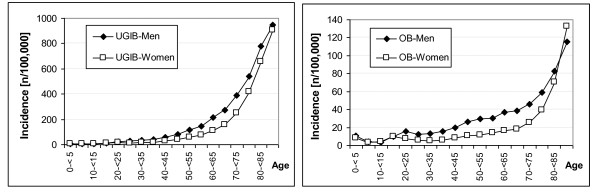
**Incidence of Gastrointestinal Bleeding Diagnoses by Age and Sex**. Sex and age-specific incidence of hospitalisations due to upper gastrointestinal bleeding (UGIB: left) and oesophageal bleeding (OB: right) in 2005 per 100,000 population.

On average, there were 16,463 hospitalisations per year due to OB (female 40.6%) and 94,232 hospitalisations per year due to UGIB (female 48.6%) over the time period 2000-2005. The incidence of hospitalisations for both outcomes changed only slightly from 2000 to 2005 (Figure 1). The sex and age-specific incidence of hospitalisations with OB and UGIB diagnoses were similar in all analysed calendar years. As an example, the incidence of hospitalisations in 2005 is presented in Figure 2. Men displayed a slightly higher incidence of hospitalisations, but in both sexes, the incidence of hospitalisations increased in a similar way with age.

**Figure 3:**
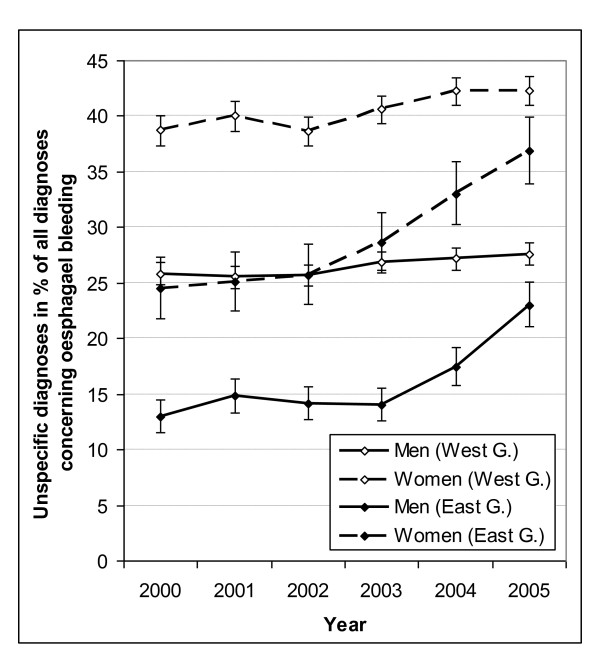
**Percentage of Unspecific Codes for Oesophageal Bleeding by Sex, Region and Year**. Cases with unspecific diagnosis in % of all cases with any diagnosis concerning hospitalisations for oesophageal bleeding for men and women in former West Germany (West G.) and former East Germany (East G.) with 95% confidence intervals (vertical bars).

Diagnostic coding for OB was more specific for men than for women, and it was also more specific in former East Germany than in former West Germany (Figure 3). This East -West difference became smaller from 2003 onwards when the proportion of unspecific codes increased in former East Germany. The change over time was parallel for both sexes in both regions.

**Figure 4:**
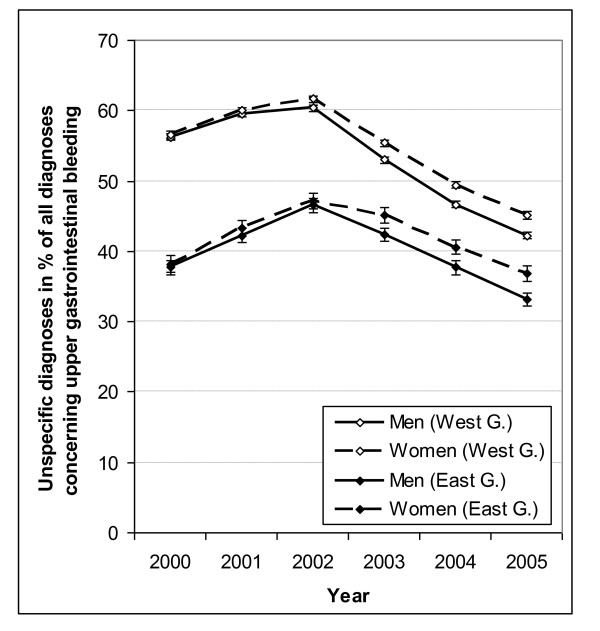
**Percentage of Unspecific Codes for Gastrointestinal Bleeding by Sex, Region and Year**. Cases with unspecific diagnosis in % of all cases with any diagnosis concerning hospitalisations for upper gastrointestinal bleeding for men and women in former West Germany (West G.) and former East Germany (East G.) with 95% confidence intervals (vertical bars: only visible if not obscured by symbols for point estimates).

Similarly, coding of UGIB diagnoses was also more specific in the former East than in former West Germany (Figure 4). There was hardly any difference in coding for men and women from 2000 to 2002, while thereafter the estimates slightly diverged towards a more specific coding in men than in women. The proportion of unspecific UGIB diagnoses increased from 2000 until 2002 in both the East and the West, while starting in 2003 a decrease in the proportion of unspecific coding was observed.

**Figure 5:**
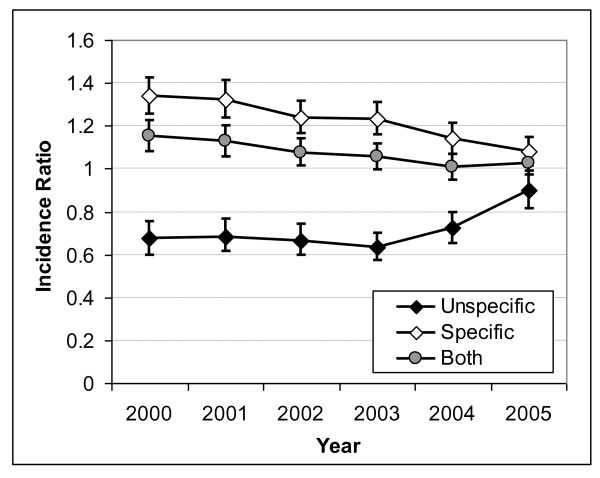
**Incidence Ratios of Specific, Unspecific and all Oesophageal Bleeding Diagnoses for former Eastern Germany compared to former Western Germany**. Incidence ratios of specific, unspecific and any coding of hospitalisations for oesophageal bleeding comparing former East Germany versus former West Germany with 95% confidence intervals (vertical bars).

**Figure 6:**
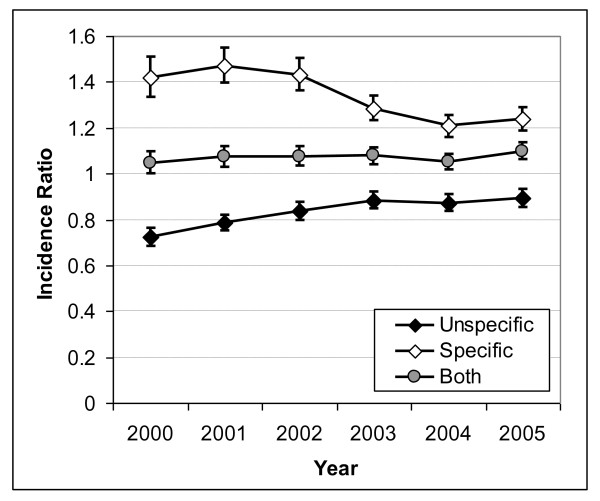
**Incidence Ratios of Specific, Unspecific and all Gastrointestinal Bleeding Diagnoses for former Eastern Germany compared to former Western Germany**. Incidence ratios of specific, unspecific and any coding of hospitalisations for upper gastrointestinal bleeding comparing former East Germany versus former West Germany with 95% confidence intervals (vertical bars).

After adjusting for sex, age and population density at the district level, the differences in hospitalisations for specific and unspecific OB and UGIB diagnoses between the East and the West remained significant (Figures 5 and 6). This East-West difference became smaller over the years, particularly from 2003 onwards. For OB, the incidence ratio of hospitalisations with specific coding in former East vs. West Germany decreased from 1.34 (95% CI 1.25-1.43) in the year 2000 to 1.08 (95% CI 1.01-1.15) in the year 2005 (Figure 5). In contrast to OB, the change in diagnostic coding over time was less marked for UGIB (Figure 6). The incidence of hospitalisations with specific coding was 42% higher in former East versus West Germany (incidence ratio = 1.42; 95% CI 1.34-1.51) in 2000, but this difference decreased to 24% in 2005 (incidence ratio = 1.24; 95% CI 1.19-1.29). For both OB and UGIB, when all diagnoses were considered, respectively, the East-West ratio was close to 1 over the studied time period, consistently with a similar overall incidence of hospitalisations in both parts of Germany. PD (data not shown) had no influence on the incidence of hospitalisations due to UGIB. This was similar for hospitalisations due to OB but with slightly increased rates for high PD versus low PD (incidence rate ratio around 1.1) for the years 2000 to 2003.

## Discussion

Health insurance claims data are an important data source to analyse incidence or the burden of disease on a population level and to conduct health services research [3,5-8,20-23]. Regional or temporal differences in the burden of disease derived from these data may reflect "true" regional or temporal differences in the incidence, but they can also be due to regional or temporal differences in coding practices. Therefore, studies conducting health services research based on hospitalisation records may be affected by coding practices. To our knowledge, variations in coding have not been investigated for Germany so far. Our results showed that overall hospitalisation rates for OB and UGIB were only marginally higher in the East than in the West. Despite this fact, the coding for OB and UGIB was more specific in former East Germany, although the differences between both regions decreased over time. The historical separation of East and West Germany might have contributed to these regional differences in coding practice.

Coding differences became smaller from 2003 onwards, especially with respect to specific and unspecific OB diagnoses. In 2003, a reimbursement system based on diagnosis related groups (DRG) was introduced for hospitals in Germany, while detailed mandatory coding rules for hospital diagnoses were first introduced in 2002. For the following years our results showed an increase in the proportion of unspecific codes for OB hospitalisations and a decrease in the proportion of unspecific codes for UGIB hospitalisations while the total incidence of hospitalisations due to OB or UGIB, respectively, remained on the same level as in the previous years.

Corresponding to this pattern Preyra [24] showed that the introduction of a complexity adjustment to a Case Mix Groups (CMG) system (which is a Canadian adaptation of DRGs) resulted in a significant increase of the hospital case mix variance without concomitant changes in morbidity or resource use. In a random sample of inpatient cases, Klaus et al. [25] estimated an overcoding in 34% of diagnoses under DRG conditions whereas undercoding was only present in 9% of diagnoses.

In Germany, in addition to reducing the costs of patient treatment and optimisation of health care, one of the intended effects was the more uniform and specific coding of hospitalisation diagnoses [26]. This effect could be seen for UGIB where the percentage of cases with unspecific coding decreased in East and West Germany for both sexes in the years following the introduction of DRGs. For OB, by contrast, we found an increase of unspecific coding in the former East after 2003, both for men and women, while in the former West the percentage of cases with unspecific coding was nearly unaffected over the same time period. The reasons for this difference and change over time are not clear. Despite a long time since the reunification of Germany, some regional variation in training of medical staff might still exist. There are also slightly different traditions in the organisation of the health care system with a more pronounced role of polyclinics for outpatient services in the former East. Still, it is not clear why these differences should result in a different coding specificity. One potential explanation for the increase in unspecific coding of OB can be the fact that the reimbursement for treatment of cases coded with unspecific bleeding diagnoses was higher than for treatment of cases coded with specific bleeding diagnoses even if the ascertainment of the bleeding cause required additional procedures (gastroscopy). In such a way, surplus services reduced the reimbursement. While we do not assume that indicated gastroscopies were not conducted, there is a possibility that unspecific coding was preferred. This problem of "reduced reimbursement while providing additional service" was recognized in 2004 but it was not immediately solved so that it persisted in 2005 (see [15], page 105). From today's perspective, it is difficult to understand the interaction between the change in coding rules and coding practices which affected the reimbursement for the hospitals. Nevertheless, our study provides evidence that such factors need to be considered. Further studies are also needed to assess the reasons for regionally different vulnerability to changes in coding rules. For Belgian hospitals, Aelvoet et al. [27] found not only that coding practices improved over time but also that there was evidence for fraudulent undercoding.

Our study is limited to the investigation of only two disease entities. We do not know whether the observed regional and temporal variations in coding also apply to other disease entities. For Germany, no systematic analysis of the coding quality and regional variations in coding quality of claims data has been published up to date [28] and published data on the quality of coding using ICD-10 are rare in general [29,30]. In Australia, Henderson et al. found a high level of reliability for principal codes of hospital discharge letters when comparing hospital coding and auditor coding [29]. However, Stausberg et al. demonstrated that the agreement in coding decreased when more detailed (for example five digit versus three digit ICD-10) codes were used and concluded that very detailed classification and complex coding rules for ICD-10 diagnoses cause significant difficulties even for coding experts [30]. The complexity and ambiguity of the coding rules may contribute to the formation of different coding preferences, which could result in the differences we observed.

Furthermore, in our analysis, it was not possible to distinguish between "true" differences in disease epidemiology and coding differences. For example, we were not able to adjust for the levels of alcohol consumption which may increase incidence of oesophageal bleeding. On the other hand, we could show that regional and temporal differences in the incidence of hospitalisations due to OB or UGIB nearly disappeared when specific and unspecific codes were analysed together. This supports the assumption that "true" disease differences were only to a minor degree accountable for the found regional and temporal differences.

## Conclusions

We found regional and temporal variations in specific and unspecific coding of the main discharge diagnosis of hospitalisations due to OB or UGIB in Germany, while there was little difference when both specific and unspecific diagnoses were considered together. Incidence and prevalence estimates of diseases based on hospitalisation diagnoses as well as results of health services research studies based on these data may be influenced by regional or temporal variations in coding of the diagnoses. Based on two disease entities, we demonstrated that regional and temporal variations in coding should be considered in the interpretation of results. Particularly, an introduction of new coding rules and changes in the reimbursement system must be considered in the study design, analysis and interpretation. Analysing a broader selection of disease entities including specific and unspecific codes will reduce the influence of varying coding practices.

## Competing interests

The authors declare that they have no competing interests.

## Authors' contributions

EG and IL conceived of the study. IL participated in developing the design of the study, performed the statistical analyses, drafted the manuscript and prepared the final version. EG participated in developing the design of the study and revised the manuscript critically. RM provided input for the interpretation of the statistical results and revised the manuscript. All authors read and approved the final manuscript.

## Pre-publication history

The pre-publication history for this paper can be accessed here:

http://www.biomedcentral.com/1472-6963/11/193/prepub
